# Dynamics of Macrophage, T and B Cell Infiltration Within Pulmonary Granulomas Induced by *Mycobacterium tuberculosis* in Two Non-Human Primate Models of Aerosol Infection

**DOI:** 10.3389/fimmu.2021.776913

**Published:** 2022-01-06

**Authors:** Laura Hunter, Suzie Hingley-Wilson, Graham R. Stewart, Sally A. Sharpe, Francisco Javier Salguero

**Affiliations:** ^1^ Research and Evaluation, UK Health Security Agency (UKHSA), Salisbury, United Kingdom; ^2^ School of Biosciences and Medicine, University of Surrey, Guildford, United Kingdom

**Keywords:** tuberculosis, pathology, macrophage, lymphocyte, rhesus macaque, cynomolgus macaque, pulmonary granuloma, immunohistochemistry

## Abstract

Non-human primate models of Tuberculosis (TB) are one of the most commonly used within the experimental TB field because they closely mimic the whole spectrum of disease progression of human TB. However, the early cellular interactions of the pulmonary granuloma are still not well understood. The use of this model allows investigation into the early interactions of cells within pulmonary granulomas which cannot be undertaken in human samples. Pulmonary granulomas from rhesus and cynomolgus macaques from two timepoints post infection were categorised into categories 1 – 6 (early to late stage granulomas) and immunohistochemistry was used to identify CD68+ macrophages, CD3+ T cells and CD20+ B cells. Multinucleated giant cells and acid-fast bacilli were also quantified. At week four post infection, cynomolgus macaques were found to have more CD68+ cells than rhesus in all but category 1 granulomas. Cynomolgus also had a significantly higher percentage of CD20+ B cells in category 1 granulomas. At week twelve post infection, CD68+ cells were most abundant in category 4 and 5 granulomas in both species; however, there were no significant differences between them. CD3+ T cells and CD20+ B cells were significantly higher in the majority of granuloma categories in cynomolgus compared to rhesus. Multinucleated giant cells and acid-fast bacilli were most abundant in categories 5 and 6 at week 12 post challenge in both species. This study has identified the basic cellular composition and spatial distribution of immune cells within pulmonary granulomas in both rhesus and cynomolgus macaques over time. The data from this study will add to the knowledge already gained in this field and may inform future research on vaccines and therapeutics for TB.

## Introduction

*Mycobacterium tuberculosis* (MTb) is part of the Mycobacterium tuberculosis complex (MTBC). The MTBC is a group of genetically related mycobacterium species that can cause tuberculosis (TB) in humans and other animals. The MTBC consists of; *M tuberculosis, M bovis, M africanum, M canettii, M microti, M mungi, M orygis, M caprae, M pinnipedii and M suricattae.* MTb is one of the leading causes of death in humans from an infectious agent worldwide and it was the number one cause of death for a single infectious agent in 2019 (1). In 2019, 1.4 million people died from TB whilst an estimated 10 million fell ill with the infection. TB is transmitted *via* aerosols when an infected person coughs, sneezes or spits (1).

Investigation of the early interactions of MTb with host cells and lesion development in humans is challenging as the moment a person became infected with TB may not be apparent for many months or even years (latent TB). Therefore, obtaining tissue samples to study early cellular interactions with the host would be very difficult as the point of infection is unknown, and the person would likely still present as healthy. Therefore, animal models are important for studying the development of disease.

Non-human primates (NHPs) have been used as a model for TB research for many decades as they are similar to humans in their anatomy, physiology and response to infection with MTb, exhibiting the whole spectrum of human disease and pathology (2,3). Rhesus macaques (*Macaca mulatta*) and Cynomolgus macaques (*Macaca fascicularis*) are the most commonly used NHP species within the UK and have a wide range of immunologic reagents available for use. It is well known that there are immunological and genetical differences between these species as well as differences in their ability to control TB, with each bringing their strengths to the TB research field (4–6). For example, rhesus macaques (RM) are usually used for vaccine evaluation studies as the outcome of infection is more uniform than cynomolgus macaques (CM), whereas CM are commonly used for drug evaluation studies as they are better able to control disease (2,3). It has been shown that RM develop a more progressive disease whilst CMs have a superior ability to control disease progression that can comparatively reduce disease burden when compared to RM (7,8). Other ‘New World’ primate species, such as the common marmoset can be used and show many aspects of human TB disease. However, studies are limited due to the lack of availability of immunologic reagents and the limited number of suppliers (2).

The pathological hallmark lesion of TB is the granuloma which primarily affects the lungs. The formation of a granuloma results from the hosts attempts at containing the invading bacteria locally by surrounding them with immune cells to prevent further spread. The classical granuloma is an organised structure made up of a variety of immune cells, typically consisting of a lymphocyte cuff on the outer edge of the lesion that surrounds a rim of macrophages which, in turn, surround a central necrotic, caseous core (9). There are various stages of development involved in the formation of a classical granuloma and these have been described and categorised in previous reports for MTb for both NHPs (10–12) and humans (13,14).

Macrophages are one of the main cellular components of a granuloma and, along with dendritic cells, are the first line of defence against MTb. However, they are not always advantageous to the host (15,16). Macrophages can produce both pro- and anti-inflammatory cytokines and chemokines, which balance the immune response between bacterial killing and host survival (17). However, if this response is not balanced, the macrophage can become a niche environment for MTb to replicate (15,16).

Within granulomas, epithelioid macrophages can fuse together to form multinucleated giant cells [MNGCs (18)]. MNGCs (or Langhan giant cells) have multiple nuclei arranged in a semi-circle and are morphologically distinct from other cells (19). The function of MNGCs within TB is still not fully understood, however, some studies have suggested that their role involves inflammation and bacterial control (20). There are limited data on MNGCs in NHP *M tuberculosis* granulomas, therefore gaining data on the number of MNGCs per granuloma type in two species of macaques at two different timepoints may provide useful information.

B and T cells are two of the other main components of a granuloma and play a central role in the adaptive immune response to MTb infection. This response is facilitated by T cells which are critical for the control of TB infection. T cells produce several cytokines and chemokines which, in turn, recruit additional immune cells to the site of infection (18,21). One such cytokine, IFN-γ, produced by Th1 helper cells, is the main mediator of macrophage activation which is important in the development of the granuloma. T cells also help to regulate the balance between pro- and anti-inflammatory immune responses (22).

The role of B cells within TB granulomas is still not fully understood. Previous studies have shown that B cells are important for the control of TB (23); are a critical component of granuloma development (24) and that at the site of infection they can regulate the host-pathogen interaction (reviewed in (25). As previously mentioned, RM and CM have differences in their ability to control TB so investigating the spatial distribution and quantifying B cells within the granuloma is important in understanding the mechanisms behind the differences.

Whilst the structure of a classical granuloma has been well defined, there is a lack of data in the NHP model with regards to the spatial distribution and quantification of immune cells within different categories of granuloma formation at different time points after infection. There is also a lack of data comparing granuloma composition in RM and CM. The comparative differences between species may provide important insights into the mechanism behind the differences in the ability to control MTb infection observed between species. These data may also be helpful to inform vaccine and therapeutic trials.

The aim of this study was to characterise immune cell populations within different granuloma stages (categories) in both RM and CM using histochemical and immunohistochemical techniques to investigate the spatial distribution of immune cells as well as quantifying the number of positive cells present in each granuloma. Data from both RM and CM were compared to identify any differences between the species. This could lead to novel immunotherapeutic strategies with which to combat human tuberculosis.

## Materials and Methods

### Experimental Animals

All formalin-fixed tissue samples used in this study were sourced from archived material collected from macaques enrolled in a study conducted at UK Health Security Agency, Porton, as previously described (8). Briefly, eight male Indian origin RM and eight male CM, aged three to four years and obtained from established UK breeding colonies, were exposed to aerosols of MTb, Erdman K01 strain containing an average presented dose calculated as 18 - 25 colony forming units (CFU) (**Table 1**). Four RM and four CM (group A) were euthanised four weeks after aerosol exposure. Another four RM and four CM (group B) were challenged using the same conditions and separately in time to group A and euthanised twelve weeks post challenge (wpc). Animals were housed in compatible social groups, in accordance with the Home Office (UK) Code of Practice for the Housing and Care of Animals Used in Scientific Procedures (1989), (now updated to Code of Practice for the housing and Care of Animals Bred, Supplied or Used for Scientific Purposes, December 2014), and the National Committee for Refinement, Reduction and Replacement (NC3Rs), Guidelines on Primate Accommodation, Care and Use, August 2006 (26). All animal procedures were approved by the UK Health Security Agency Ethical Review Committee, Porton Down, UK, and authorised under an appropriate UK Home Office project license.

**Table 1:** Experimental parameters and granuloma category breakdown for all animals analysed.

Animal ID	Species	Examination week post infection	Presented exposure dose [CFU]	Disease burden measure		Number of granulomas counted						
							Granuloma categories					
				Total pathology score	Pulmonary Lesion count	Total	1	2	3	4	5	6
T7	Rhesus	4	24	10	10	75	47	20	2	–	6	–
T21	Rhesus	4	24	9	15	72	49	10	–	5	8	–
T26	Rhesus	4	25	16	7	60	40	11	–	5	3	–
T41	Rhesus	4	25	7	27	65	48	11	1	1	4	–
980BEEA	Cynomolgus	4	25	6	17	17	12	3	–	–	2	–
978AJB	Cynomolgus	4	23	8	12	19	12	5	1	1	–	–
970CIA	Cynomolgus	4	27	8	9	28	17	2	3	2	4	–
545ACEA	Cynomolgus	4	25	12	44	10	9	–	–	–	1	–
T59	Rhesus	12	25	15	2	32	26	2	–	2	2	–
T75	Rhesus	12	24	21	10	95	55	16	–	16	8	–
U22	Rhesus	12	18	40	51	148	11	26	5	55	46	5
U15	Rhesus	12	19	29	1401	68	5	11	9	7	19	17
044HAFC	Cynomolgus	12	23	15	22	29	19	6	–	2	1	1
031MN	Cynomolgus	12	28	9	3	23	21	1	–	–	–	1
406ADM	Cynomolgus	12	24	15	7	84	34	10	–	18	19	3
980ABAGB	Cynomolgus	12	23	12	13	17	15	1	–	–	–	1

Total pathology score = gross pathology. Pulmonary lesion count = whole lung gross count. Granulomas counted = right upper and left lower lung lobes only.

### Histopathology

At necropsy, lungs were removed intact and fixed by intra-tracheal infusion with 10% neutral buffered formalin [NBF] (Solmedia Ltd, Shrewsbury, UK) as previously described (8). Once inflated, the lungs were immersed in 10% NBF until required for processing. Representative samples from each lung lobe (left upper, middle and lower lung lobes and right upper, middle, lower and accessory lung lobes) containing visible lesions (where present) were processed to paraffin wax blocks using standard histological procedures, sectioned at 4µm and stained with haematoxylin and eosin (HE) for routine pathological evaluation. The aerosol route of MTb infection has been shown to distribute the challenge dose evenly throughout all of the lung lobes of the animal (27–29) which allowed the analysis for immune cell composition to be concentrated on two representative sections of lung lobe per animal rather than all seven lobes. Consecutive sections from archived tissue blocks of the right upper and left lower lung lobes from each animal were sectioned and stained by immunohistochemistry (CD68, CD3, CD20) and the Ziehl-Neelsen (ZN) technique to identify acid fast bacilli (AFB).

**Figure 1:**
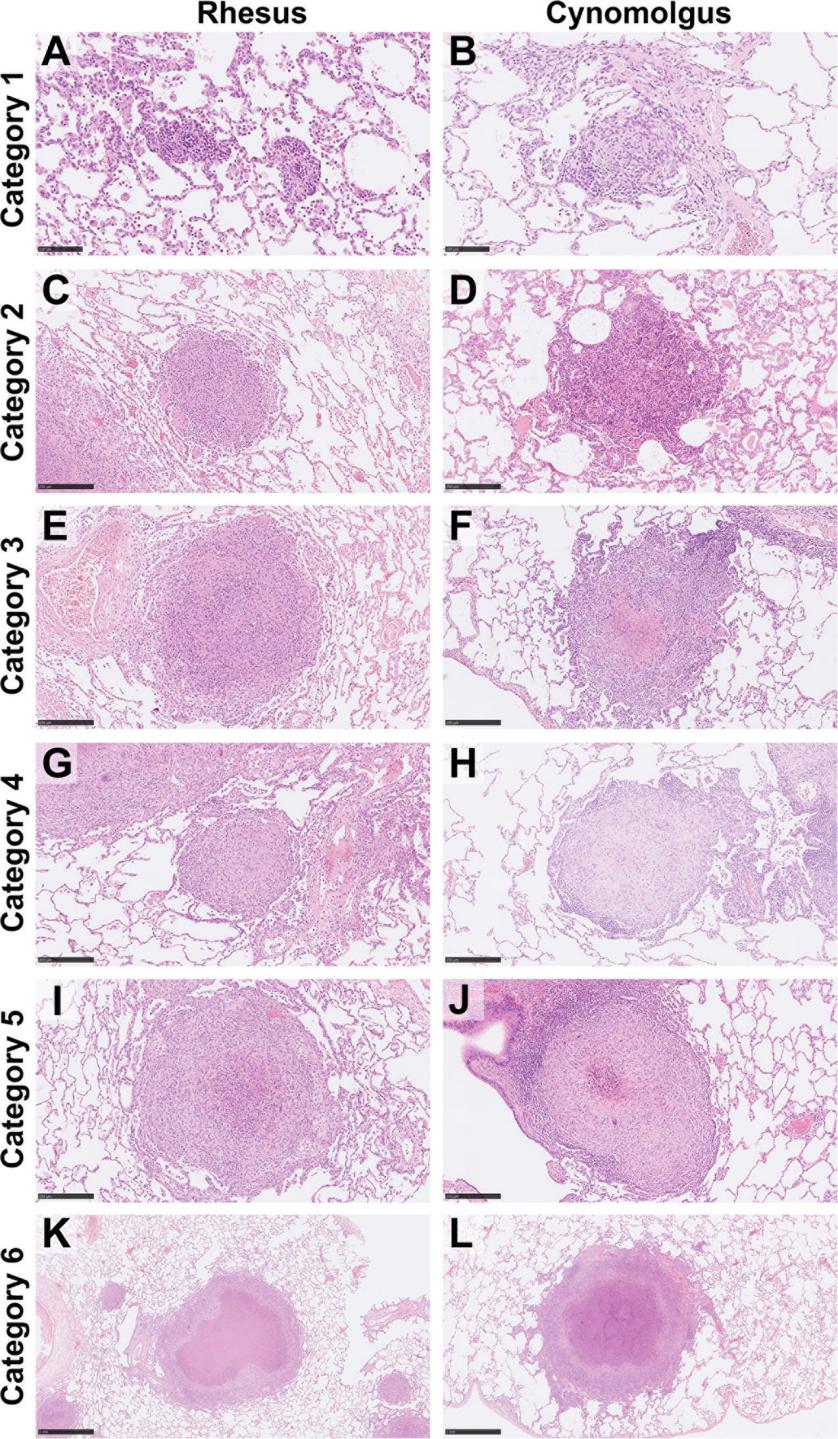
Representative images of each granuloma category (1–6) for both rhesus and cynomolgus macaques. HE. Category 1 **(A, B)** granulomas are small, unorganised, diffuse foci of mixed inflammatory cells but lack a peripheral cuff of lymphocytes. Category 2 **(C, D)** granulomas consist of similar cells types as category 1 lesions but are larger and more defined, becoming circular in shape. Category 3 **(E, F)** granulomas are the same as category 2 but with the addition of focal necrosis. Category 4 **(G, H)** granulomas are an organised structure that are well circumscribed and consist mainly of macrophages and evidence of a few peripheral lymphocytes. Category 4 lesions that exhibit necrotic foci with degenerate neutrophils are classed as category 5 **(I, J)**. Classic, largely well-demarcated granulomas with central, caseous necrosis and a variable rim of lymphocytes are classified as category 6 **(K, L)**. Bars in micrographs **(A, B)**= 100µm, **(C–J)** = 250µm, **(K, L)** = 1000µm.

A granuloma scoring system was used, as previously described by Rayner et al. (10) and Sibley et al. (29) to identify, categorise and count microscopic lesions in lung sections from all NHPs in groups A and B (**Figure 1** and **Table 2**).

**Table 2:** Granuloma category descriptions. Modified from Rayner et al. (10).

Granuloma category	Organised or unorganised	Necrotic or non-necrotic	Description
1	Unorganised	Non-necrotic	Small, diffuse foci consisting of macrophages, lymphocytes, scattered neutrophils, and eosinophils that lack clearly defined boundaries.
2	Unorganised	Non-necrotic	Similar inflammatory cell types as category 1 lesions but are larger and are more defined.
3	Unorganised	Necrotic	Lesions similar to category 2 but with the addition of focal necrosis, defined by pyknosis and karyorrhexis with the loss of cellular architecture.
4	Organised	Non-necrotic	Well circumscribed, organised lesions which consist primarily of macrophages admixed with neutrophils and other leucocytes with evidence of few peripheral lymphocytes.
5	Organised	Necrotic	Lesions similar to category 4 lesions with the addition of a necrotic foci with degenerate neutrophils.
6	Organised	Necrotic	Classical, largely well-demarcated lesion with central, caseous necrosis and a variable rim of lymphocytes.

Unorganised = lacking peripheral lymphocyte cuff; organised = peripheral lymphocyte cuff present.

Numbers of AFB and multinucleated giant cells (MNGC) were quantified by microscopy from ZN stained slides as previously described (30). The total number of bacteria present in each granuloma were counted. Numbers of AFB were recorded using a scale of 0 – 3 (0 = no bacilli, 1 = 1 – 10, 2 = 11 – 50 and 3 ≥ 50 AFBs). The total number of MNGCs identified in each granuloma were counted and scored on a scale of 0 -2 (0 = no MNGC, 1 = 1 – 10 and 2 = ≥ 10 MNGCs).

### Immunohistochemistry

Sections from the right upper lung lobe and left lower lung lobe from each animal were cut at 4 µm, placed on SuperFrost slides and stained to identify B cells (CD20), T cells (CD3) and macrophages (CD68) within lung lesions using the Leica ‘Bond RXm’ and ‘BOND polymer refine’ detection kit with horseradish peroxidase (HRP) to visualise the cell markers. Sections were dewaxed in xylene, rehydrated through alcohol, and distilled water and treated in 3% hydrogen peroxide (five minutes) to quench endogenous peroxidase activity. Depending on the antibody used, tissue sections (one antibody per tissue section) were pre-treated for antigen retrieval by heat-induced epitope retrieval on the Leica ‘Bond RXm’ automated immunostainer using either Leica Epitope retrieval solution 1 (pH 6) or Leica Epitope retrieval solution 2 (pH 9) for either; 20 minutes (anti-CD68 – ER1 and anti-CD3 – ER2) or 30 minutes (anti-CD20 – ER1). After antigen retrieval, anti-CD3 (rabbit polyclonal anti-human, product code: A0452, Agilent, Santa Clara, CA) was used at a dilution of 1:200 with an incubation time of 15 minutes, whereas anti-CD68 (mouse monoclonal anti-human, Clone KP1, product code: M0814, Agilent) and anti-CD20 (Mouse monoclonal, anti-human, Clone L26, product code: M0755, Agilent) first had a universal blocking step (Superblock TBS blocking buffer, Thermo Fisher Scientific, product code: 37581, Loughborough, UK) for 20 minutes before the antibodies were incubated for 30 mins (anti-CD68; 1:400) and 15 minutes (anti-CD20; 1:150). All antibodies were incubated at ambient temperature on the ‘Leica Bond RXm’. A post primary rabbit anti mouse IgG (eight minutes) followed by an anti-rabbit Poly-HRP-IgG (eight minutes. Both included in the Leica Polymer Refine kit) were applied. DAB was used as the chromogen for visualisation and sections were counterstained with Harris’ haematoxylin for ten minutes (Lecia Bond Polymer Refine detection kit).

### Image Analysis

Right upper and left lower lung lobe slides stained by immunohistochemistry were scanned with a ‘NanoZoomer S360’ (Hamamatsu, Japan), viewed and images taken using ‘NDP.View 2’ software (version 2.8.24, Hamamatsu Photonics K.K, Japan). Digital image analysis was performed using ‘Nikon NIS elements AR’ software (version 5.01.00, Nikon, Amsterdam, Netherlands). All granulomas within each tissue section were analysed. In total, for each species at each timepoint eight slides were analysed (two per animal). The whole area of the granuloma was selected as a ‘Region of Interest’ (ROI), and the area with immunohistochemically positive reaction within the ROI was calculated by the software after setting the thresholds. The results were expressed as the percentage of positively stained areas within the total area of the granuloma. Necrotic or mineralised areas were not included in the ROI to be analysed.

### Statistical Analysis

Median percentage of positive cells for CD68+, CD20+ and CD3+ staining in tissue sections collected from RM and CM at both timepoints were compared using Mann-Whitney’s U test between categories and timepoints. Numbers of AFBs and MNGCs between both species, granuloma categories and at both time points were analysed using a one-way ANOVA followed by Tukey’s multiple comparison test (GraphPad Prism 7.0, GraphPad Software, San Diego, CA, USA). Correlations between multiple variables were analysed using Pearson correlation coefficients (Jamovi for macOS, version 1.6.23). In all analyses, a *P* value of <0.05 was considered significant.

## Results

**Figure 2:**
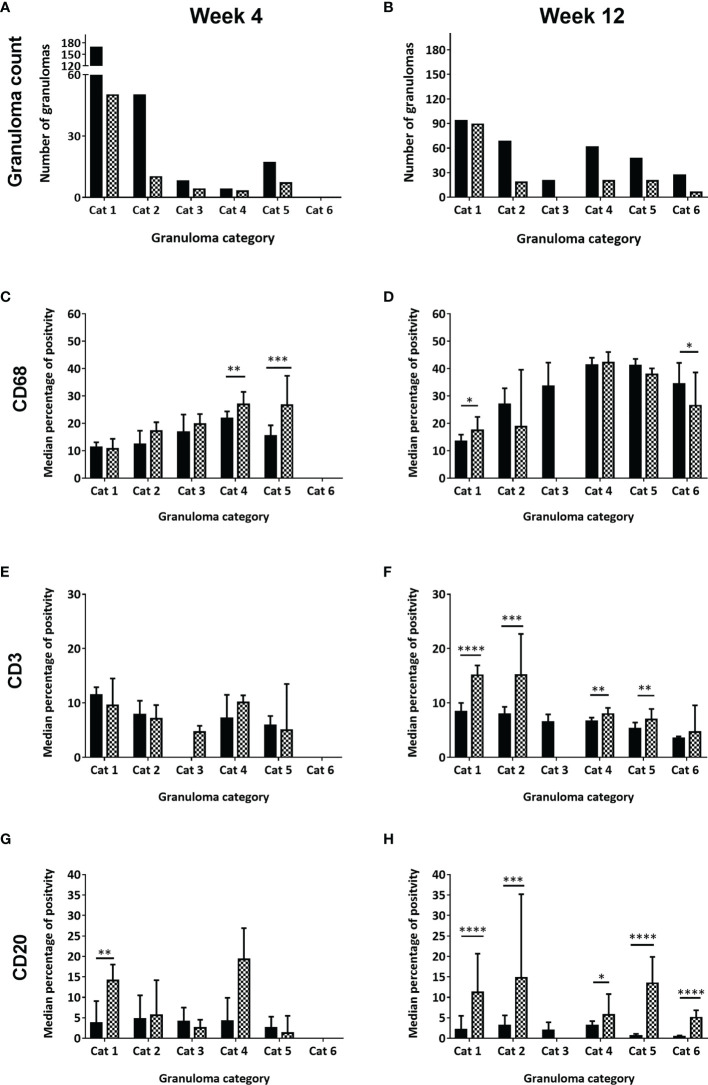
Number of granulomas observed, scored and counted and the median percentage of positively stained immune cells (CD68+, CD3+, CD20+) in each granuloma category at week four and week twelve post challenge, for rhesus and cynomolgus macaques. Total number of granulomas counted in both rhesus and cynomolgus macaques at 4wpc **(A)** and 12wpc **(B)**. Black column represents rhesus macaques (4 per time point) and checked column represent cynomolgus macaques (4 per time point). Median percentage of positive cells in all granulomas counted in each category at 4wpc and 12wpc for: CD68+ macrophages **(C, D)**, CD3+ T cells **(E, F)** and CD20+ B cells **(G, H)**. Mann Whitney U test, bars represent median values, error bars represent 95% confidence interval. *P < 0.05, **P < 0.01, ***P < 0.001, ****P = < 0.0001.

Granulomas from all categories were identified in both RM and CM (**Figure 1** and **Table 2**). At 4 wpc and at 12 wpc, category 1 granulomas were the most abundant type in both RM and CM (**Figures 2A, B**). At 4 wpc (**Table 1**), there was a trend for the number of lung granulomas in RM to be greater than the number identified in CM, with category 1 and 2 granulomas making up the majority of all the granulomas in the lung sections evaluated. Granulomas of categories 3 and 4 had the lowest counts, while category 6 granulomas were not observed at 4 wpc in either species (**Figures 2A, B**). At 12 wpc (**Table 1**), category 1 granulomas were still the most abundant type in both species (**Figures 2A, B**). Each granuloma was then further characterised *via* cell type and number of AFB and MNGCs.

### CD68+ Macrophages

**Figure 3:**
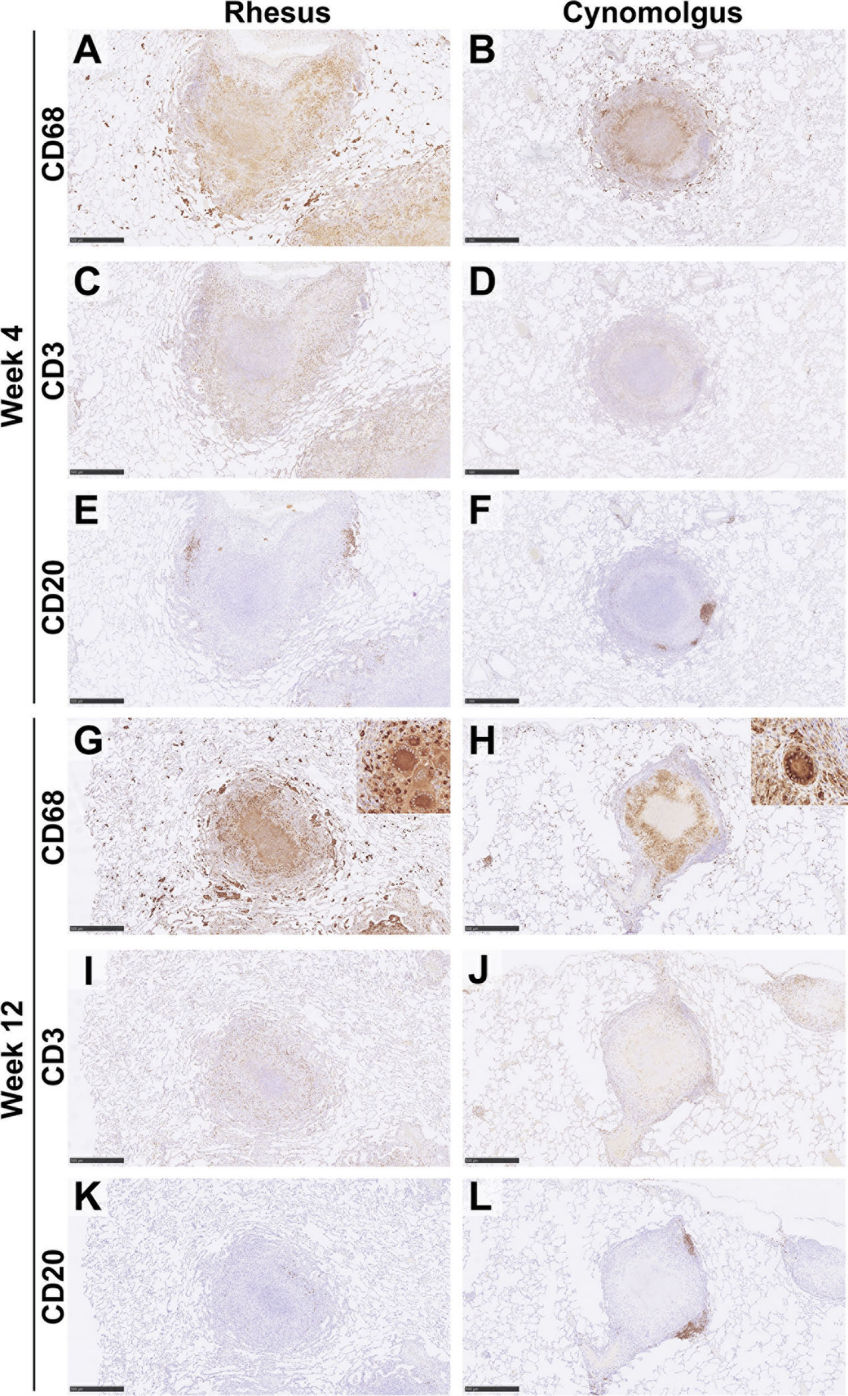
Representative images of immune cell staining (brown) in category 5 granulomas at 4wpc and 12wpc in rhesus and cynomolgus macaques. IHC. Images show the distribution of immune cells in a category 5 granuloma from RM and CM. At 4wpc, CD68+ cells were predominately confined to the centre of the granuloma and form a cuff of cells separating the lymphocyte cuff from necrosis **(A, B)**. CD3+ T cells were found scattered in a lymphocyte cuff on the periphery of the granuloma **(C, D)**. CD20+ B cells are predominately found in follicle like structures on the periphery of the granuloma **(E, F)**. At 12wpc, location of cells were similar to 4wpc [CD68+ **(G, H)**; CD3+, **(I, J)**], however CD20+ B cells in RM do not always form follicle like structures **(K, L)**. Bars in micrographs **(A, C, E, G–L)** = 500µm, **(B, D, F)** = 1000µm. Representative images from 2 slides per animal at each time point (week 4 & 12 = 4 RM & 4 CM). See **Supplementary Material** for images of all categories at each time point in both RM & CM.

CD68 is a member of the scavenger receptor family and is expressed by macrophages including epithelioid macrophages and MNGCs. Macrophages were the main cellular component of all granuloma category types (**Figure 3**). In categories 1 -3 (unorganised) macrophages were observed scattered throughout the granuloma; by contrast they were located predominately in the centre of category 4 (organised) granulomas and formed a rim between the necrotic centre and lymphocyte cuff in categories 5 and 6 (organised) granulomas (**Table 2**).

Overall, an increasing trend in the number of CD68+ macrophages from category 1 to category 5 granulomas was observed, with the highest numbers noted in the latter (**Figure 2**). At 4 wpc (**Figures 2C**, **3A**), a higher number of CD68+ macrophages were present in granuloma types from CM compared to those from RM in all but category 1 granulomas where numbers were equal in both species. However, the only differences between the species that reached statistical significance were found in category 4 (*P*=0.0082) and category 5 (*P*=0.0007) granulomas. At 12 wpc (**Figures 2D**, **3B**), CD68+ cells appeared to be most abundant in category 4 and 5 granulomas, but differences between the species did not reach significance. However, significant differences were seen in category 1 granulomas with CM possessing a higher percentage of CD68+ cells (*P*=0.0152) and in category 6 granulomas where a higher percentage were noted in RM compared to CM (*P*=0.0166).

### CD3+ T Cells

CD3 is part of the TCR complex and is a pan T cell marker. Lymphocytes stained with anti-CD3 were found scattered throughout granulomas in categories 1 – 3, whilst in categories 4 – 6 they were arranged in a cuff around the outside edge of the granuloma (**Table 2**). At 4 wpc (**Figures 2E**, **3C**), a higher percentage of CD3+ T cells were present in category 1 & 2 granulomas in RM compared to CM. Category 3 granulomas were not identified in pulmonary tissue sections from RM at 4 wpc. CM had a higher percentage of CD3+ T cells in category 4 compared to RM; however, the opposite was observed in category 5 granulomas where RM granulomas contained more CD3+ T cells than those in CM. Category 6 granulomas were not identified at 4 wpc in either RM or CM. Overall, the number of CD3+ T cells did not differ between RM and CM at 4 wpc. At 12 wpc (**Figures 2F**, **3D**) CD3+ T cells were more abundant in granulomas classified in categories 1 and 2 compared to those in categories 4, 5 and 6 in both species. A significantly higher percentage of CD3+ T cells was identified in category 1 (*P*=<0.0001), category 2 (*P*=0.0001), category 4 (*P*=0.0081) and category 5 (*P*=0.0025) granulomas from CM compared to those from RM. A nonsignificant trend for the percentage of CD3+ T cells to be higher in category 6 granulomas from CM compared to RM was observed. Category 3 granulomas were not identified in CM and were therefore excluded from the comparative analysis.

### CD20+ B Cells

CD20 is a transmembrane protein which is found on B cells throughout their development, from pre-B cells through to terminal differentiation into plasma cells. Similar to T cells, CD20+ B cells tended to be scattered throughout the granuloma in the unorganised granuloma categories (1 – 3, **Table 2**). In the organised granuloma categories (4 – 6), CD20+ B cells were often arranged in follicular-like structures on the periphery of the granulomas (**Figure 3**, week 4 E & F: week 12 K & L). At 4 wpc (**Figure 2G**), the percentage of B cells in category 1 granulomas was significantly higher (*P*=0.0017) in CM than RM. In categories 3 and 5, RM had a higher percentage of CD20+ B cells compared to CM, whilst the opposite trend was observed in categories 2 and 4. Category 6 granulomas were not observed at 4 wpc in either RM or CM. At 12 wpc (**Figure 2H**), overall, more CD20+ B cells were found in CM, with differences reaching significance in categories 1 (*P*=<0.0001), 2 (*P*=0.0002), 4 (*P*=0.0168), 5 (*P*=<0.0001) and 6 (*P*=<0.0001). Category 3 could not be analysed statistically as these were not observed in CM samples at 12 wpc.

### Acid Fast Bacilli

**Figure 4:**
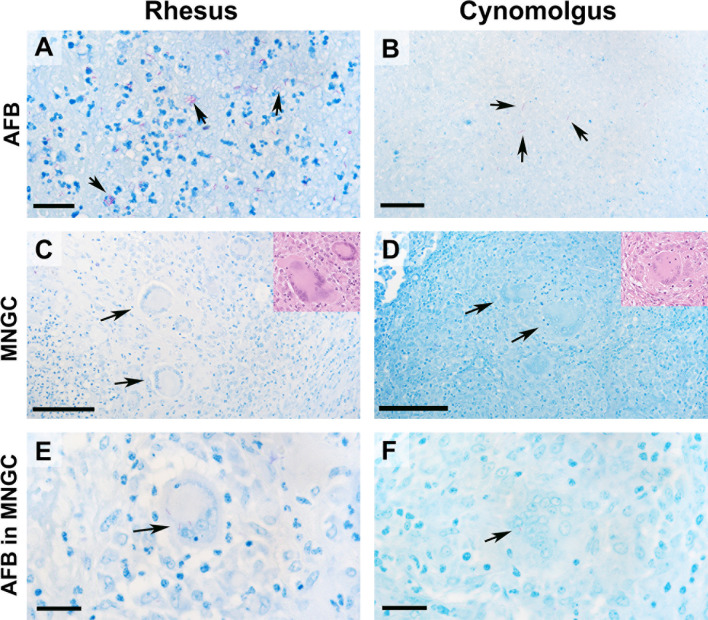
AFB bacilli were most abundant in category 6 granulomas in both rhesus and cynomolgus macaques. Representative images of AFB staining and multinucleated giant cells ZN (inserts HE). AFB were most abundant in category 6 granulomas in both species **(A, B)**. In RM, AFB were frequently seen grouped together whereas AFB in CM were more often seen singularly within the necrotic core. MNGCs were identified in both species **(C, D)** with 12wpc category 6 granulomas having the highest number. RM on average had a higher proportion of MNGCs. AFBs were noticed within MNGCs in both species, although not frequently **(E, F)**. Bars in micrograph = 200µm. Representative images from 2 slides per animal at each time point (week 4 & 12 = 4 RM & 4 CM).

In organised, necrotic lesions (categories 5 and 6, **Table 2**) AFB were extra-cellular and observed within necrotic debris around the edge of the necrotic centre, whereas in category 4 lesions, as well as in the unorganised lesions (categories 1 – 3), AFB were observed scattered throughout the granuloma. In both species, AFB were seen within MNGCs (**Figures 4E, F**). In RM, AFB were not observed in category 1 granulomas but were present in categories 2 - 5 at 4 wpc, whilst at 12 wpc, AFB were only observed in granulomas classified as categories 3 – 6 (**Table 3**). In CM at 4 wpc, AFB were observed in categories 1 & 2 and 4 – 5, however they were only observed in categories 5 and 6 at 12 wpc.

**Table 3:** Descriptive statistics of acid-fast bacilli counts for each category of granuloma stage.

Granuloma Category	Rhesus						Cynomolgus					
	Week 4			Week 12			Week 4			Week 12		
	Number of observations	Mean ± SD	Range	Number of observations	Mean ± SD	Min/Max Range	Number of observations	Mean ± SD	Range	Number of observations	Mean ± SD	Min/Max Range
1	49	0	0	27	0	0	21	0.05 ± 0.22	0 – 1	26	0	0
2	32	0.19 ± 0.59	0 – 3	55	0	0	17	0.06 ± 0.24	0 – 1	45	0	0
3	2	1.50 ± 2.12	0 – 3	14	0.43 ± 1.34	0 – 5	0	N/A	N/A	9	0	0
4	11	0.27 ± 0.65	0 – 2	43	0.35 ± 1.13	0 – 6	7	0.14 ± 0.38	0 – 1	27	0	0
5	17	17.47 ± 20.16	0 – 51	52	2.56 ± 8.85	0 - 51	8	13.00 ± 17.73	0 – 51	38	0.45 ± 0	0 – 5
6	0	N/A	N/A	23	11.43 ± 20.27	0 - 51	0	N/A	N/A	8	22 ± 24.54	0 - 51

Number of observations = number of granulomas counted in all granuloma categories at each time point. Range = range of AFB observed in granulomas. Mean = mean number of AFB observed ± SD.

A significantly higher number of AFB were counted in the category 5 granulomas than in granulomas in the lower categories examined in RM 4 weeks after challenge; categories 1 and 5 (*P*=<0.0001), categories 2 and 5 (*P*=<0.0001) and categories 4 and 5 (*P*=<0.0001) (**Table 3**). At the equivalent time point post challenge in CM, significantly higher numbers of AFB were also found in category 5 granulomas compared to the lower categories of granulomas; categories 1 and 5 (*P*=0.0001), categories 2 and 5 *(P*=0.0002*)* and categories 4 and 5 (*P*=0.0029) (**Table 3**). Comparison of the granuloma categories between species did not show any difference in the amount of AFB counted. Twelve weeks after challenge, examination of granulomas from RM revealed that category 6 granulomas had significantly higher numbers of AFB than early stage categories (1 – 4); categories 1 and 6 (*P*=0.0316), categories 2 and 6 (*P*=0.0092) and categories 4 and 6 (*P*=0.0262) (**Table 3** and **Figure 4A**). At the same time point in CM, category 6 granulomas were observed to have a significantly higher number of AFB than all other categories; categories 1 and 6 (*P*=<0.0001), categories 2 and 6 (*P*=<0.0001), categories 3 and 6 (*P*=<0.0001), categories 4 and 6 (*P*=<0.0001) and categories 5 and 6 (*P*=<0.0001) (**Table 3** and **Figure 4B**). Comparison of AFB numbers in granulomas of each category between species did not identify any differences. At both time points and in both RM and CM, numbers of AFB were significantly higher in granulomas with a necrotic centre (categories 5 and 6) compared to granulomas with non-necrotic centres (categories 1, 2 and 4).

### Multinucleated Giant Cells

MNGCs in organised, necrotic lesions were observed around the edge of the necrotic core and into the lymphocytic rim, whereas, in category 4 granulomas, MNGCs were generally noted in the centre of the granuloma. In the unorganised lesions, MNGCs were seen scattered within the granuloma. MNGCs were not observed in category 1 granulomas in either RM or CM.

MNGCs were observed in both species and were most abundant twelve weeks after challenge when MNGCs were present in most granuloma categories (**Figures 4C, D** and **Table 4**). At 4 wpc, MNGCs were only noted in granulomas from RM in categories 2 and 5 with similar numbers counted (**Table 4**). At 12 wpc, MNGCs were found in categories 2 – 6 and the numbers increased as the granulomas developed (**Table 4**). In CM at 4 wpc, MNGCs were seen only in category 5 granulomas with an average of one MNGC per granuloma. At 12 wpc, MNGCs were seen in categories 2 – 6 with categories 5 and 6 having the most MNGCs.

**Table 4:** Descriptive statistics of multinucleated giant cells (MNGCs) counts for each category of granuloma stage.

Granuloma Category	Rhesus						Cynomolgus					
	Week 4			Week 12			Week 4			Week 12		
	Number of observations	Mean ± SD	Range	Number of observations	Mean ± SD	Range	Number of observations	Mean ± SD	Range	Number of observations	Mean ± SD	Range
1	49	0	0	27	0	0	21	0	0	26	0	0
2	32	0.06 ± 0.35	0 – 2	55	0.42 ± 2.01	0 – 14	17	0	0	45	0.11 ± 0.49	0 – 3
3	2	0	0	14	2.36 ± 3.43	0 – 11	0	N/A	N/A	9	0.44 ± 0.88	0 – 2
4	11	0	0	43	3.30 ± 4.70	0 – 23	7	0	0	27	1.22 ± 1.72	0 – 5
5	17	0.35 ± 0.70	0 – 2	52	5.38 ± 10.54	0 – 63	8	0.88 ± 0.99	0 – 2	38	2.58 ± 4.86	0 – 29
6	0	N/A	N/A	23	17.13 ± 39.56	0 – 157	0	N/A	N/A	8	8.75 ± 7.52	3 – 23

Number of observations = number of granulomas counted in all granuloma categories at each time point. Range = range of MNGCs observed in granulomas. Mean = mean number of MNGC observed ± SD.

Four weeks after challenge, significantly higher numbers of MNGCs were observed in category 5 granulomas compared to categories 1 and 2 granulomas from RM: categories 1 and 5 (*P*=0.0025) and categories 2 and 5 (*P*=0.0354) (**Table 4**). The same observation was made in CM at the same time point where MNGC numbers were significantly higher in category 5 granulomas compared to categories 1, 2 and 4; categories 1 and 5 (*P*=<0.0001), categories 2 and 5 (*P*=<0.0001) and categories 4 and 5 (*P*=0.0002) (**Table 4**). Four weeks after challenge the number of MNGCs in granulomas of each category was similar between species; by contrast, at week twelve post challenge, categories 5 and 6 granulomas (organised with necrotic centres) from both RM and CM possessed significantly higher numbers of MNGCs.

Twelve weeks after challenge, significantly higher numbers of MNGCs were observed in category 6 granulomas compared to all other granuloma categories in RM; categories 1 and 6 (*P*=0.0004), categories 2 and 6 (*P*=<0.0001), categories 3 and 6 (*P*=0.0273), categories 4 and 6 (*P*=0.0026) and categories 5 and 6 (*P*=0.0132) (**Table 4** and **Figure 4C**). Examination of CM at the same time point revealed that category 5 and 6 granulomas (organised, necrotic centres) had significantly higher numbers of MNGCs than categories 1 and 2 (unorganised, non-necrotic, early stage of development). Furthermore, MNGC numbers were significantly higher in category 6 granulomas compared to categories 3 and 4; categories 1 and 5 (*P*=0.0138) categories 1 and 6 (*P*=<0.0001), categories 2 and 5 (*P*=0.0043), categories 2 and 6 (*P*=<0.0001), categories 3 and 6 (*P*=<0.0001), categories 4 and 6 (*P*=<0.0001) and categories 5 and 6 (*P*=<0.0001) (**Table 4** and **Figure 4D**). Comparison of the number of MNGCs present in different granuloma categories between species showed that category 4 granulomas in RM had significantly higher numbers of MNGC compared to CM (*P*=0.0211).

### Correlation Data

A correlation matrix using Pearson’s r coefficient was performed to investigate correlations between AFB score, MNGC score, granuloma categories, time (weeks post challenge) and species (**Table 5**). Positive correlations were found between; AFB and MNGC scores (*r* = 0.367, *P*=0.001), granuloma categories and AFB (*r* = 0.478, *P*=0.001), granuloma categories and MNGC scores (*r* = 0.606, *P*=0.001), time and MNGC (*r* = 0.347, *P*=0.001) and granuloma category and time (*r* = 0.300, *P*=0.001). A weak correlation was also seen between time and species (*r* = 0.089, *p*=0.041).

**Table 5:** Correlation matrix.

		Bacteria score	MNGC score	Granuloma category	Time	Species
Bacteria score	Pearson’s r	–				
	P-value	–				
MNGC score	Pearson’s r	**0.367**	–			
	p-value	**<.001**	–			
Granuloma category	Pearson’s r	**0.478**	**0.606**	–		
	p-value	**<.001**	**<.001**	–		
Time	Pearson’s r	-0.041	**0.347**	**0.300**	–	
	p-value	0.345	**<.001**	**<.001**	–	
Species	Pearson’s r	-0.076	-0.067	-0.029	**0.089**	–
	p-value	0.081	0.124	0.498	**0.041**	–

Values in bold show positive correlations.

## Discussion

In this study, the cellular composition of MTb-induced pulmonary granulomas in RM and CM was investigated using a combination of histopathology, chromogenic immunohistochemistry, and digital image analysis in order to evaluate immune cell populations. Published data quantifying the cellular components of pulmonary granulomas at different stages of disease development in the NHP TB model using chromogenic immunohistochemistry is limited and data comparing granuloma development and quantification of cellular components at different time points in both RM and CM samples is particularly sparse. For the first time, we have compared the cellular components of granulomas at different stages of development in RM and CM in a time course study. The animals were challenged by the aerosol route of infection, which has been shown to distribute the challenge dose evenly throughout the lung lobes of the animal (27–29) allowing analyses to be applied to representative lung lobes (right upper and left lower). These data will assist the detailed understanding of disease progression in the context of the comparison of disease controlling and susceptible host immune responses.

TB granulomas have been characterised in a number of animal species following infection by a range of mycobacteria species; NHPs, MTb (10–12), guinea pigs, MTb (31), cattle, *Mycobacterium bovis* (20,32), deer, *M bovis* (30) as well as humans, *M tuberculosis* (13,33). RM and CM are the most commonly used NHP models for TB research as they share the greatest number of anatomical and physiological similarities with humans, compared to other animal models (3). The whole spectrum of human TB-induced disease and pathology is seen in RM and CM and evaluation of the immune response is greatly assisted as some immunological reagents created for use within human tissues cross react with both macaque species (2,3). It is known that there are differences between the macaque species in the ability to control disease progression with RM showing a higher rate of progression and a higher level of bacterial burden compared to cynomolgus macaques of Asian origin (4,7,8,27,34). In this study RM were found to have a larger number of pulmonary granulomas compared to CM at 4 wpc and 12 wpc, although the distribution of lesions across the granuloma categories was similar in both species of NHP. We observed a higher percentage of category 6 granulomas in RM compared with CM suggesting that the disease process occurred at an accelerated rate compared to that in CM. Furthermore, the number of CD68+ cells in category 4 and 5 granulomas in CM was significantly higher than that in RM at 4 wpc and also higher than the number of CD68+ cells present at 12 wpc in RM. These data would suggest that granulomas in CM are not necrotising as quickly as those in RM, and provides further evidence that RM show a higher rate of disease progression and CM are able to control the disease more effectively.

In this study, granulomas were classified into six categories according to a system developed for NHPs by Rayner et al. (10) which described the development from early unorganised to well-developed organised granulomas with necrotic and caseous centres. Organised granulomas made up a smaller percentage of the lesions counted at 4 wpc and category 6 granulomas were not observed which was not surprising as a category 6 granuloma is the most developed form of lesion which requires longer to develop. At 12 wpc, category 1 granulomas remained the most abundant granuloma category in both RM and CM whilst the remaining number of granulomas in categories 2 – 6 were lower and similar between species.

Our data shows that the spatial distribution of T and B cells within granulomas from both species of macaques is similar to that observed by Phuah et al. (23) in their studies of CMs. T and B cells in organised lesions (categories 4 – 6) from both RM and CM were observed in an outer rim of lymphocytes around a layer of macrophages before the necrotic core. B cells also formed follicle-like structures in the lymphocyte cuff in these organised lesions and this has been observed not only in NHPs, but also in humans and bovine TB (33,35). In unorganised, smaller lesions (categories 1 – 3) T and B cells are found scattered throughout the granuloma.

Macrophages are the first line of defence against TB where they ingest the bacteria which produces an inflammatory response in pulmonary tissue, resulting in the production of pro-inflammatory cytokines and chemokines to be produced (17). The expression of these cytokines and chemokines induce the recruitment of further immune cells, namely monocytes, neutrophils and primed T and B cells, to the site of infection. During TB inflammation macrophages fuse together during granuloma progression to create MNGCs (18). The role of MNGCs in the granuloma structure is still not fully understood and some studies have suggested that MNGCs role is inflammation and bacterial control (20). MNGCs have been reported as generally localised at the peripheral epithelioid rim of the granuloma (20) and our data would agree with this statement for organised, necrotic granuloma categories. In the organised granuloma categories, MNGCs were observed in the peripheral epithelioid rim that surrounded the necrotic core whilst MNGCs were seen scattered through smaller lesions (categories 2-3) and most abundant in the centre of the non-necrotic, organised lesions, category 4. MNGCs were most abundant at 12 wpc in both RM and CM with very few observed at 4 wpc.

Our data found a trend for the number of macrophages in a granuloma to increase as the granulomas developed (from categories 1 – 5 at 4 wpc and categories 1 – 4 at 12 wpc) in both RM and CM. Also, we observed that CM had a higher number of CD68+ cells at week four post challenge when compared to RM and this is in line with a study by Sibley et al. (36) which evaluated monocytes circulating in the peripheral blood and also showed that CM’s had a higher proportion when compared to RM. These data suggest that the initial macrophage response in CMs is more effective than compared to RMs who have a lower number of macrophages at 4 wpc.

In this study, anti-CD3 was used to identify all T cells although this marker is unable to differentiate between T helper cells and cytotoxic T cells. At 12 wpc, the granulomas examined from CM possessed a significantly higher number of CD3+ T cells than those from RM. This observation is in line with the findings from an evaluation of CD3+ T cells in the mononuclear cells of peripheral blood from different macaque species (37). This study demonstrated that, in general, CMs possessed a higher proportion of CD3+ cells than RM. However, this study had evaluated CD3+ T cells collected on a number of different occasions after exposure to different challenge doses combined together for analysis. Our data suggests that at 12 weeks post infection, with a low challenge dose, CD3+ T cells follow the same trend as Sibley et al. (37) in that CMs have a higher proportion of CD3+ cells when compared to RMs. The higher proportion of CD3+ T cells would suggest that CMs were initiating a more effective T cell response compared to RM and this may be contributing to their ability to control the disease more effectively.

The role of B cell in TB granulomas is still not fully understood, however, previous studies in macaques have shown that for granulomas to be fully protective to the host, the B and T cell interaction is a critical component of granuloma development (24). Previous studies have highlighted that B cells are important for the control of TB (23) and our data substantiates this as we observed a higher proportion of CD20+ B cells, in granulomas collected from CM at both four and twelve weeks after infection compared to those from RM which show a higher disease burden and are considered to be progressors of disease. Four weeks after challenge we observed a trend for granulomas from CM to have had a higher percentage of CD20+ cells in the majority of granuloma categories (1, 3 and 4) compared to RM, whilst at 12 wpc CM had a higher percentage of CD20+ cells in all granuloma categories (1, 2, 4, 5 and 6. Category 3 granulomas were not counted at this time point in CM). In both species, CD20+ B cells were observed making follicle-like structures on the periphery of many of the granulomas in the organised lesions categories (4 – 6), this is similar to what has been reported previously (23,33,35). Our findings are in contrast with those of Fuller et al. (38) who state that they only observed these follicle-like aggregates in caseous granulomas in CM.

Another key aspect to take into account was the number of AFB present in each granuloma. We noted that AFB were mainly observed in necrotic granuloma categories (5) at 4 wpc in both species whilst at 12 wpc, in RM, bacilli were observed in granuloma categories 3 – 6 (both unorganised and organised granulomas); by contrast in CM bacilli were not observed until necrotic granuloma categories 5 and 6. These data would support the hypothesis that CM have a lower rate of disease progression compared to RM.

The main limitation in this study was sample size; we interrogated materials collected from eight rhesus and eight cynomolgus macaques at two timepoints (four RM and four CM at each time point). As this exploratory study was conducted as a pilot study to provide proof of concept for an ultra-low dose aerosol challenge the sample size is small. To add strength to this study, further data should be collected from similar studies of both species of macaque at the same timepoints.

This study has identified the basic composition of granulomas induced following infection with the Erdman strain of MTb, together with the spatial distribution of immune cells in the granuloma and changes over time in both RM and CM. Further work is now required to characterise the cytokines and chemokines produced by the different cell populations within the granulomas, and to map the spatial location of the cells expressing them. Further analysis of where AFB are present within cells or extracellularly and their spatial location could also be performed. This would add to the knowledge already gained in this field and would allow visualisation of where these interactions are occurring within the granuloma; this may inform future research on vaccines and therapeutics for TB and could lead to novel immunotherapeutic strategies with which to combat human tuberculosis. The characterisation of the different cellular populations within a granuloma will add to the knowledge of the immunological differences between RM and CM and will allow future understanding to ensure that the right model is selected for the right purpose.

## Data Availability Statement

The original contributions presented in the study are included in the article/**Supplementary Material**. Further inquiries can be directed to the corresponding author.

## Ethics Statement

The animal study was reviewed and approved by UK Health Security Agency, Animal Welfare and Ethical Review body, Porton Down, UK and authorised under an appropriate UK Home Office project license.

## Author Contributions

FS, SS, LH, SH and GS contributed to the conception and design of the study. LH performed the staining and analysis of the stained slides. All authors worked on the interpretation of the results. LH wrote the first draft of the manuscript and all authors approved the final version. All authors contributed to the article and approved the submitted version.

## Funding

This work has been funded by the Department of Health (UKHSA) and the University of Surrey.

## Conflict of Interest

The authors declare that the research was conducted in the absence of any commercial or financial relationships that could be construed as a potential conflict of interest.

## Publisher’s Note

All claims expressed in this article are solely those of the authors and do not necessarily represent those of their affiliated organizations, or those of the publisher, the editors and the reviewers. Any product that may be evaluated in this article, or claim that may be made by its manufacturer, is not guaranteed or endorsed by the publisher.

## Abbreviations

AFB, Acid fast bacilli; ANOVA, Analysis of Variance; CFU, Colony forming units; CM, Cynomolgus macaque; DAB, 3,3’-Diaminobenzidine; ER, Epitope retrieval; HE, Haematoxylin and Eosin; HRP, Horse radish peroxidase; IHC, Immunohistochemistry; MNGC, Multi-nucleated giant cells; MTb, *Mycobacterium Tuberculosis;* MTBC, Mycobacterium tuberculosis complex; NBF, Neutral buffered formalin; NC3Rs, National Centre for Replacement, Refinement and Reduction of Animals in Research; NHP, Non-human primate; RM, Rhesus macaque; ROI, Region of Interest; TB, Tuberculosis; TBS, Tris buffered saline; TCR, T-cell receptor; WHO, World Health Organisation; WPC, Week post challenge; ZN, Ziehl Neelsen.
