# Supplementary material for: Dynamics of Macrophage, T and B Cell Infiltration Within Pulmonary Granulomas Induced by Mycobacterium tuberculosis in Two Non-Human Primate Models of Aerosol Infection
Source: Front Immunol. 2022 Jan 6;12:776913. doi: 10.3389/fimmu.2021.776913 (PMC8770544; doi:10.3389/fimmu.2021.776913)
Supplement: Supplementary file 1 [file DataSheet_1.pdf]

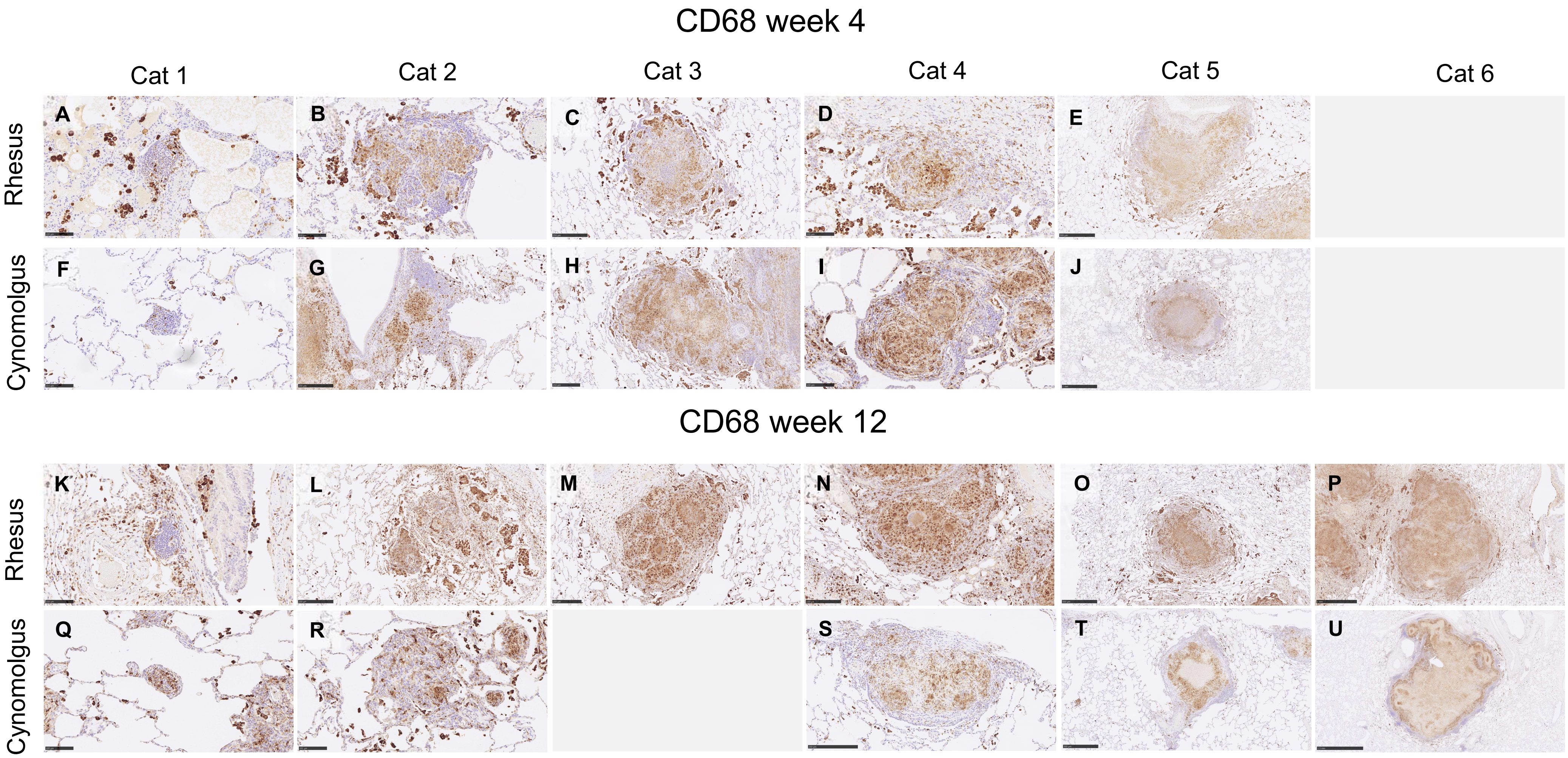

Supplementary figure 1. Representative images of CD68+ staining in all categories of granulomas in both rhesus and cynomolgus macaques at 4 and 12 wpc. Light grey boxes = no granulomas of that category were observed. Bars in micrographs – A, B, D, F, I, K, Q & R = 100µm; C, G, H, L, M, N & S = 250µm; E, O & T = 500µm; J & P = 1000µm; U = 2500µm.

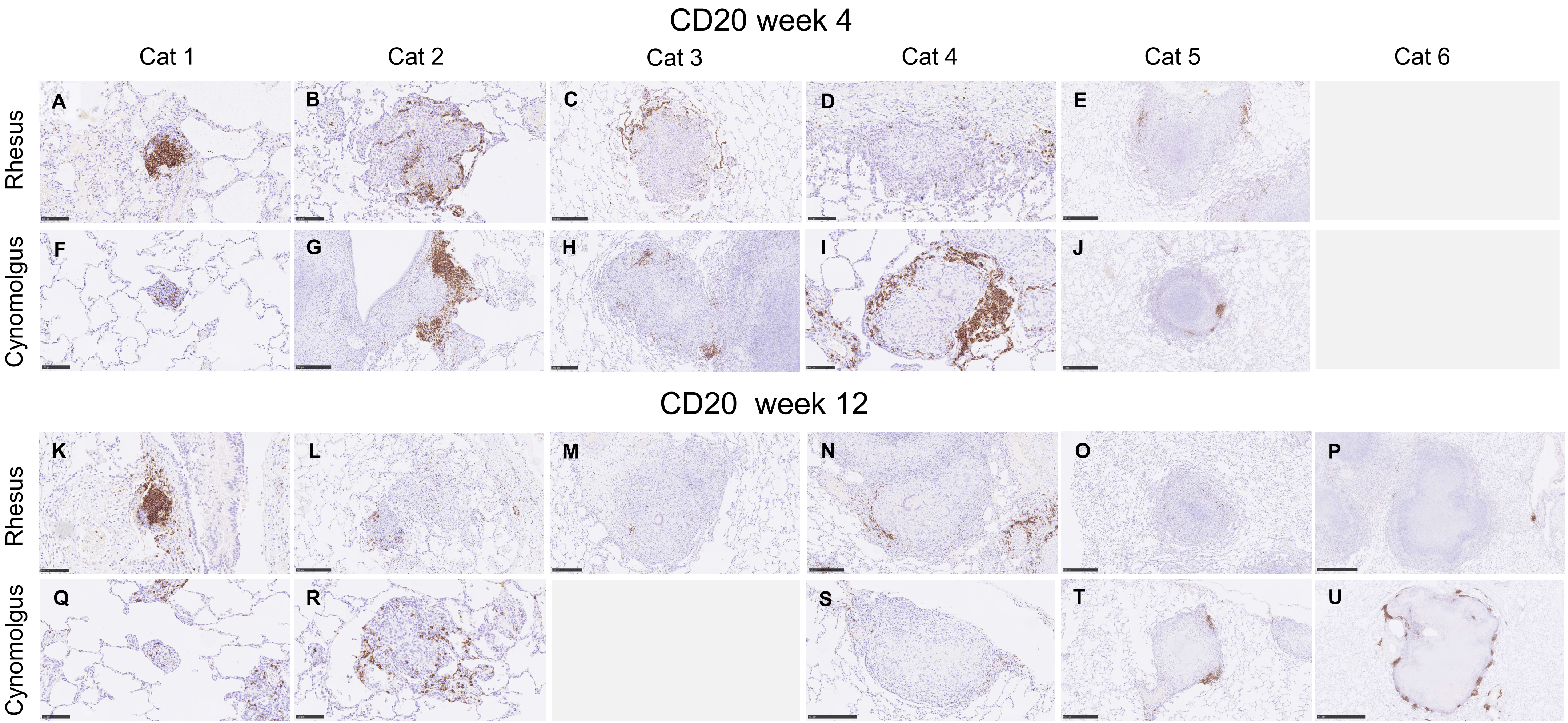

Supplementary figure 2. Representative images of CD20+ staining in all categories of granulomas in both rhesus and cynomolgus macaques at 4 and 12 wpc. Light grey boxes = no granulomas of that category were observed. Bars in micrographs – A, B, D, F, I, K, Q & R = 100µm; C, G, H, L, M, N & S = 250µm; E, O & T = 500µm; J & P = 1000µm; U = 2500µm.

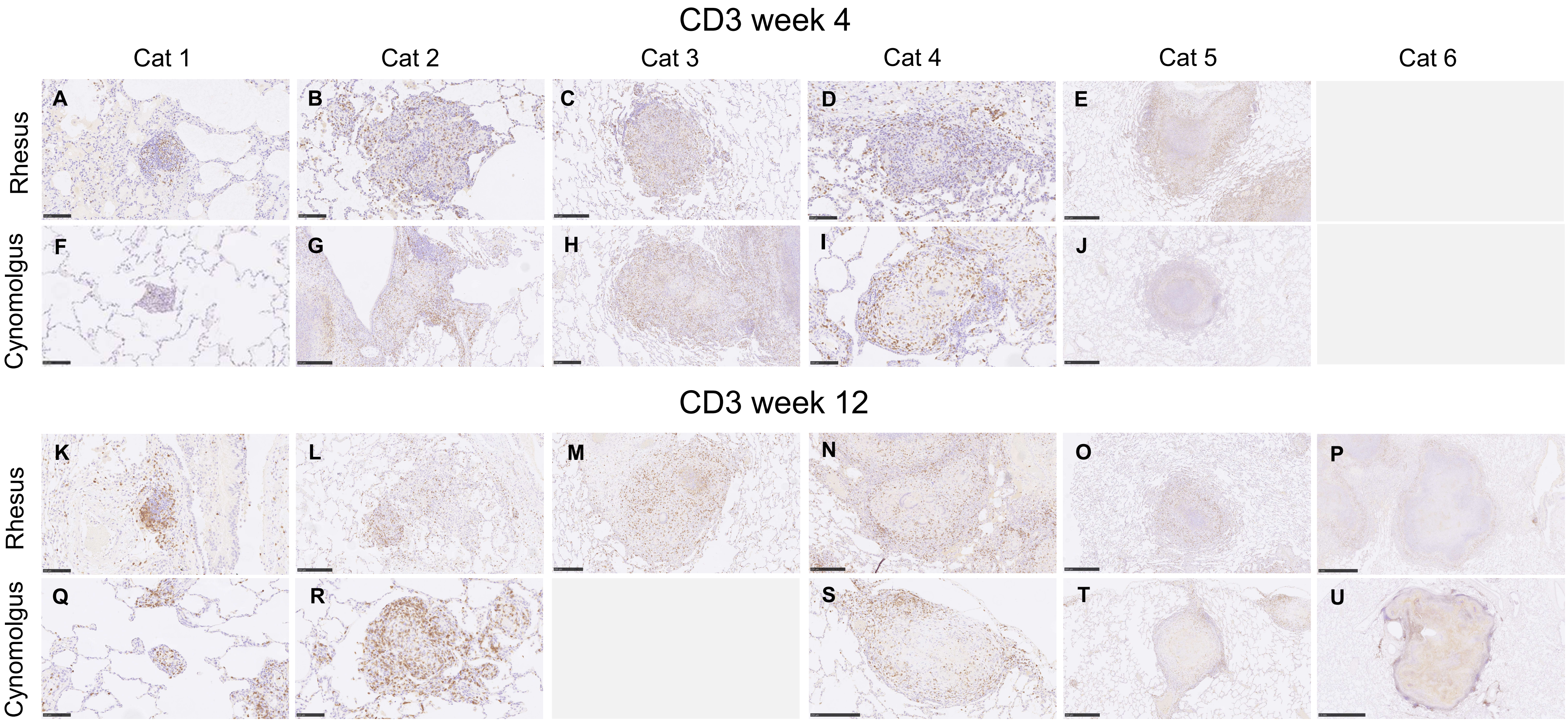

Supplementary figure 3. Representative images of CD3+ staining in all categories of granulomas in both rhesus and cynomolgus macaques at 4 and 12 wpc. Light grey boxes = no granulomas of that category were observed. Bars in micrographs – A, B, D, F, I, K, Q & R = 100µm; C, G, H, L, M, N & S = 250µm; E, O & T = 500µm; J & P = 1000µm; U = 2500µm.
